# Tractography for Optic Radiation Preservation in Transcortical Approaches to Intracerebral Lesions

**DOI:** 10.7759/cureus.1722

**Published:** 2017-09-28

**Authors:** Vijay Agarwal, James G Malcolm, Gustavo Pradilla, Daniel L Barrow

**Affiliations:** 1 Department of Neurosurgery, Emory University School of Medicine; 2 Department of Neurological Surgery, Emory University School of Medicine

**Keywords:** neurosurgery, meningioma, diffusion tensor imaging, synaptive, brainpath, intraventricular mass

## Abstract

We present a case of intraventricular meningioma resected via a transcortical approach using tractography for optic radiation and arcuate fasciculus preservation. We include a review of the literature.

A 54-year-old woman with a history of breast cancer presented with gait imbalance. Workup revealed a mass in the atrium of the left lateral ventricle consistent with a meningioma. Whole brain automated diffusion tensor imaging (DTI) was used to plan a transcortical resection while sparing the optic radiations and arcuate fasciculus. A left posterior parietal craniotomy was performed using the Synaptive BrightMatter™ frameless navigation (Synaptive Medical, Toronto, Canada) to minimally disrupt the white matter pathways. A gross total resection was achieved. Postoperatively, the patient had temporary right upper extremity weakness, which improved, and her visual fields and speech remained intact. Pathology confirmed a World Health Organization (WHO) Grade I meningothelial meningioma.

While a thorough understanding of cortical anatomy is essential for safe resection of eloquent or deep-seated lesions, significant variability in fiber bundles, such as optic radiations and the arcuate fasciculus, necessitates a more individualized understanding of a patient’s potential surgical risk. The addition of enhanced DTI to the neurosurgeon’s armamentarium may allow for more complete resections of difficult intracerebral lesions while minimizing complications, such as visual deficit.

## Introduction

Intraventricular meningiomas are quite rare, accounting for less than 5% of all intracranial meningiomas [1]. As opposed to meningiomas found in other intracranial compartments, they do not have dural attachments and instead arise from the stroma of the choroid plexus or the tela choroidea. Distribution within the ventricles is reported to be 77.8% in the lateral ventricle (usually in the region of the atrium), 15.6% in the third ventricle, and 6.6% in the fourth ventricle [2]. Treating these tumors has posed a unique challenge for neurosurgeons. They often remain clinically silent until they have reached a significant size. Also, due to the difficult location, an expert knowledge of cortical anatomy, meticulous planning, and sound microsurgical technique is required to minimize the morbidity of these operations. With the advent of intraoperative tools, such as stereotactic guidance, clinical outcomes continue to improve; however, visual complications and speech deficits remain a mainstay in the treatment of these lesions.

In this study, we review a case of a dominant hemisphere intraventricular meningioma treated via a transcortical approach with the assistance of enhanced automated whole-brain diffusion tensor imaging (DTI) in preoperative planning for the avoidance of the visual pathways and the arcuate fasciculus. We also review the literature on approaches to intraventricular lesions with the use of tractography for trajectory and intraoperative planning to avoid visual compromise.

## Case presentation

A retrospective review was conducted of a patient who underwent a transcortical approach for surgical resection of a primary intraventricular tumor. Whole brain automated diffusion tensor imaging was utilized for surgical planning and approach purposes using the Synaptive BrightMatter™ navigation system (Synaptive Medical, Toronto, Canada). Surgical records, histologic records, and imaging studies were analyzed.

**Figure 1:**
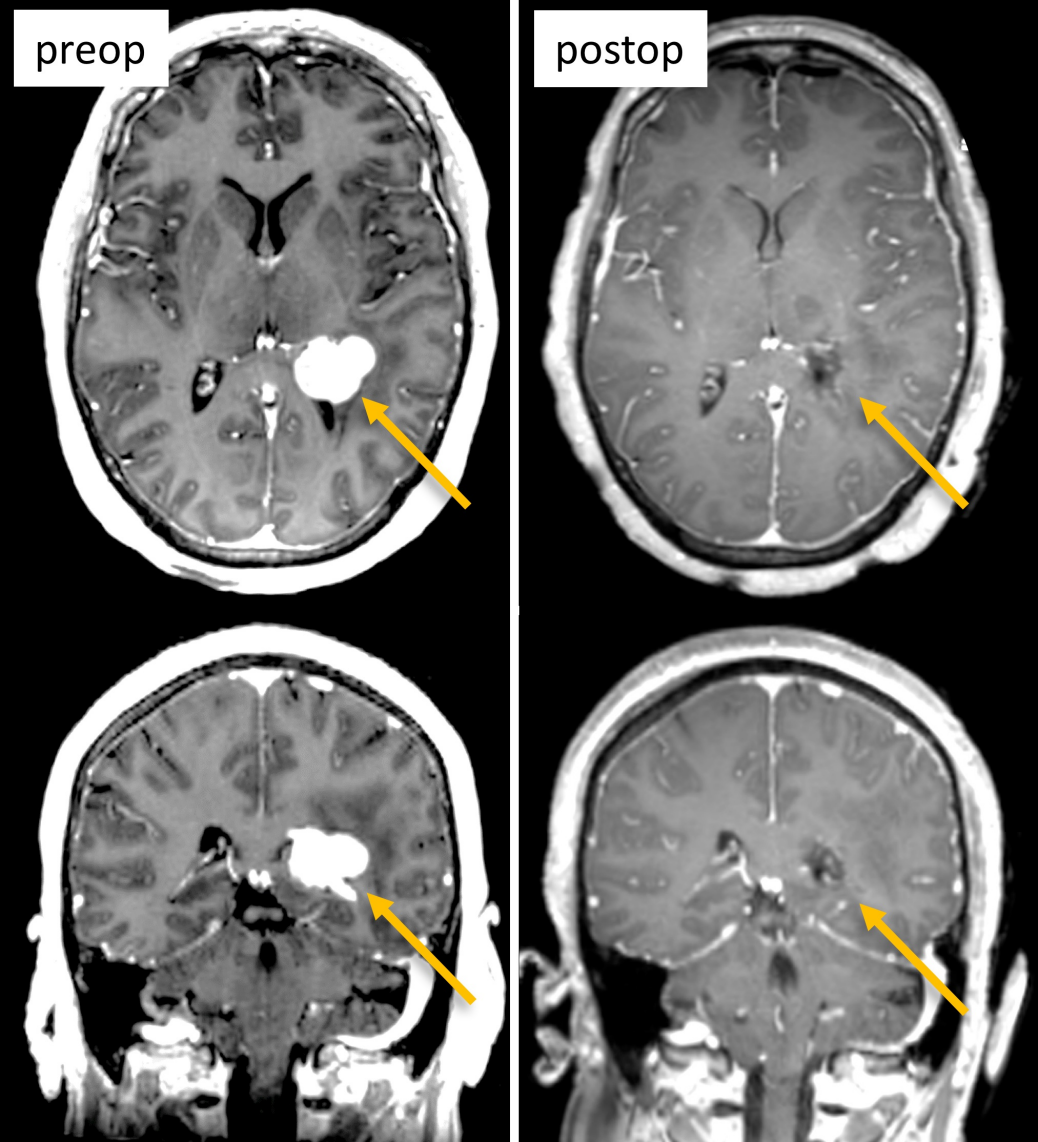
Pre- and postoperative MRI (T1 with contrast, axial top row, coronal bottom row) showing the lesion in the apex of left lateral ventricle (left column, arrows) and gross total resection (right column, arrows). MRI: magnetic resonance imaging

The case is of a 54-year-old woman with a history of breast cancer who noted gait imbalance, which began two years previously. As part of this workup, a magnetic resonance image (MRI) of the brain demonstrated a homogeneously enhancing lesion in the atrium of the left lateral ventricle, presumed to be a meningioma. The remaining workup was negative. The patient was offered treatment at that time but elected to observe and follow the lesion. More recently, she developed increasing gait imbalance, short-term memory loss, and personality changes. Repeat MRI showed growth of the lesion (Figure 1). On physical exam, the patient did not show any neurological deficit. On formal ophthalmological exam, Humphrey visual fields were normal and she scored 14/14 (100%) on detection of Ishihara color plates. After the risks and benefits of surgical intervention were discussed, the patient consented to a left parietal craniotomy with a transcortical approach and tumor resection.

**Figure 2:**
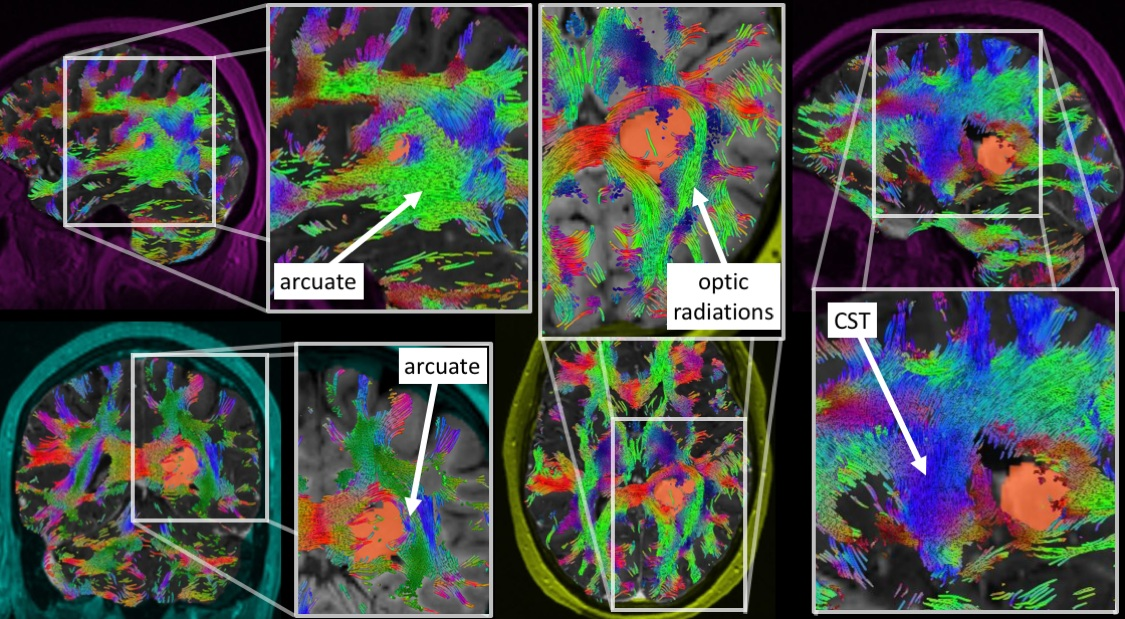
Projected views of tumor (orange) with surrounding white matter pathways (colors): acuate fasciculus, optic radiations, and corticospinal tract (CST) The tumor is bordered laterally by the optic radiations and arcuate, which prevents certain operative corridors.

**Figure 3:**
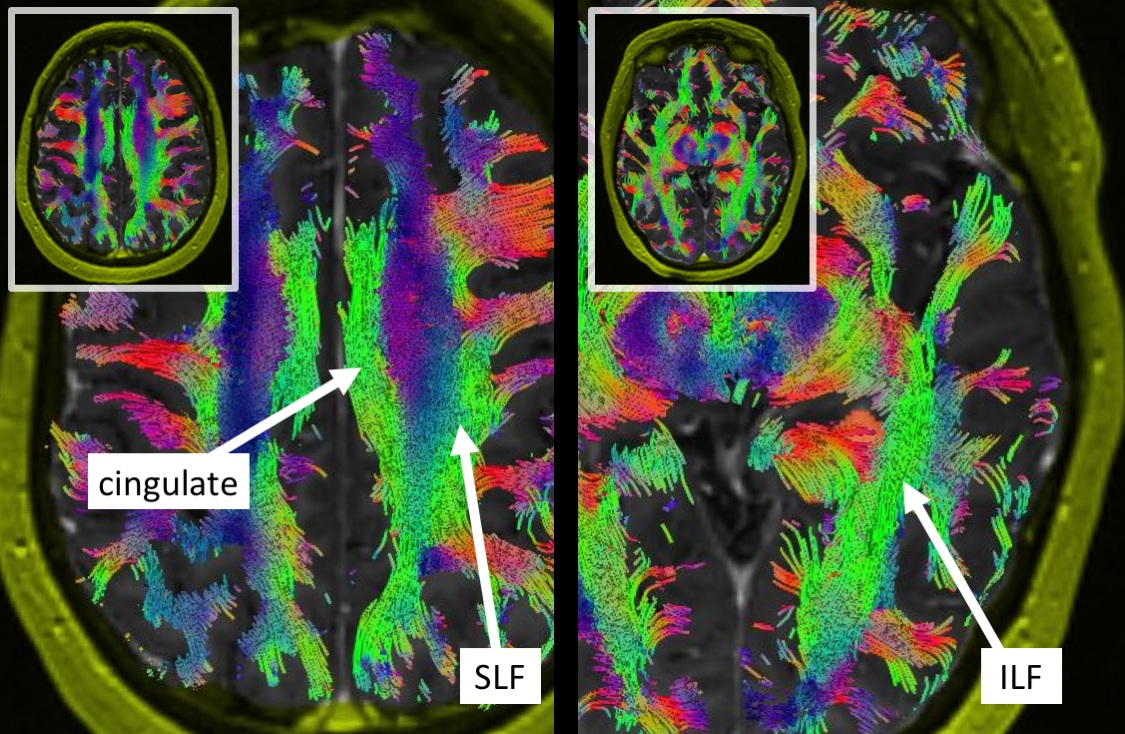
Axial views of the long tracts bordering the tumor: cingulate, superior longitudinal fasciculus (SLF), and inferior longitudinal fasciculus (ILF). The SLF makes a superior-parietal approach difficult.

**Figure 4:**
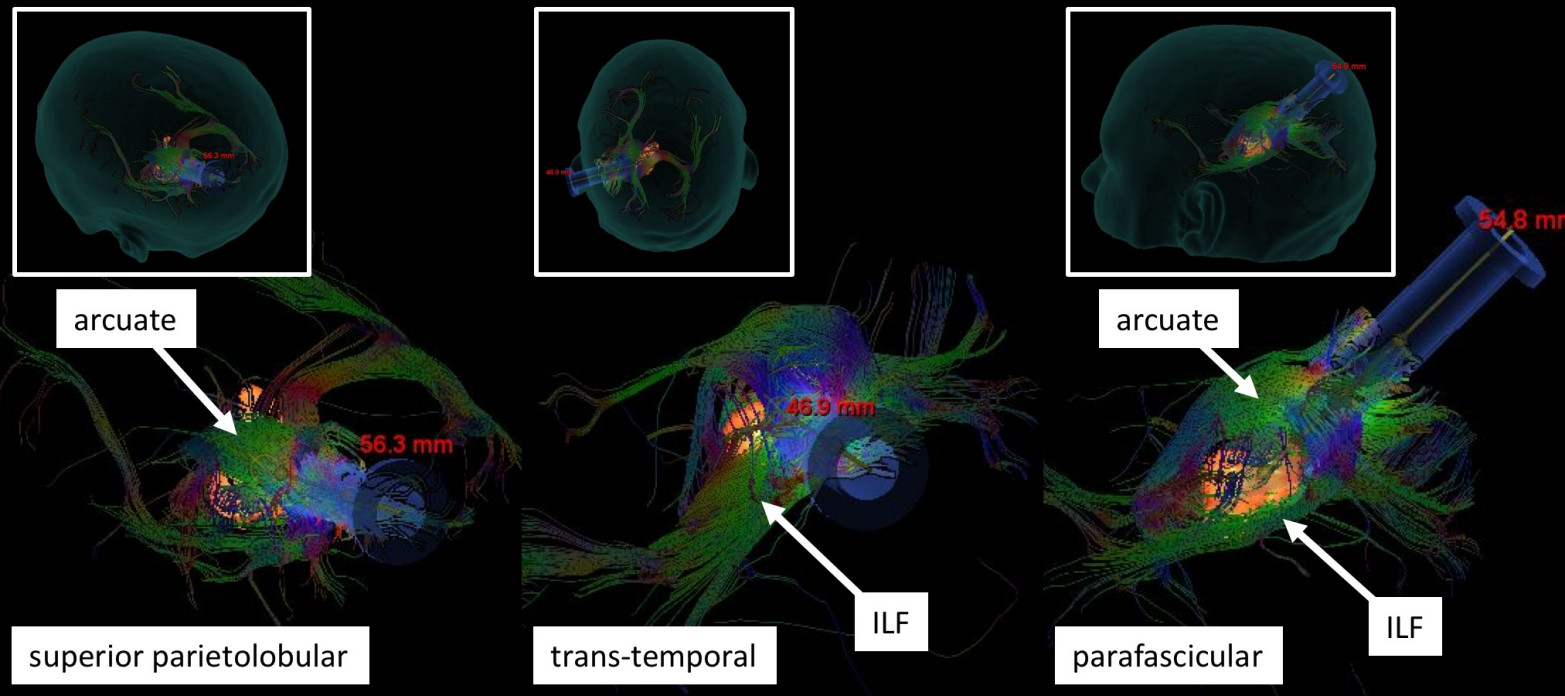
Three candidate trajectories. The superior parieto-lobular trajectory (left) risks damaging the arcuate fasciculus. The transtemporal trajectory (middle) risks damaging the inferior longitudinal fasciculus (ILF). The parafascicular trajectory (right) was ultimately chosen for surgery, which allowed access between the arcuate and ILF with minimal disruption.

Traditional transtemporal and superior parietal lobule trajectories were simulated using BrightMatter™, but the trajectories would cause transection of critical white matter fibers. Figure 2 projects several views of the tumor (orange) surrounded by important tracts. From these views, we can see the tumor was bordered laterally and superiorly by the arcuate fasciculus, which limited a transparietal approach to only specific corridors. Figure 3 projects axial views of the superior longitudinal fasciculus (SLF) superior to the tumor and inferior longitudinal fasciculus (ILF) inferolateral to the tumor. The superior parieto-lobular approach (Figure 4, left) and transtemporal approach (Figure 4, middle) seemed likely to damage the arcuate and ILF. Ultimately, the best trajectory was parafascicular, which appeared to cause the least amount of white matter transection (Figure 4, right).

The patient was positioned supine with the head in a neutral position. A left posterior parietal craniotomy was performed using Synaptive BrightMatter™ frameless navigation. The dura was opened in a U-shaped fashion and flapped medially. The sulcal entry site was confirmed with the intraoperative navigation system. A BrainPath™ cannula probe (Nico Corporation, Indianapolis, IN) was stereotactically inserted transcortically into the atrium of the left lateral ventricle. The stylet was removed and the tumor was immediately visible under high magnification. The tumor capsule was cauterized, opened sharply, and then internally debulked with ultrasonic aspiration. The tumor capsule was cauterized and folded internally. Tumor arachnoid/capsule adhesions to the ventricular wall were further cauterized and divided, allowing the remainder of the tumor mass to be removed through the cannula. A visual gross total resection was achieved. An external ventriculostomy catheter was placed.

Postoperatively, the patient was extubated without issue. She did have some temporary right upper extremity weakness, which improved rather quickly over a few days. Her visual fields were full. Postoperative MRI of the brain showed a complete resection of the left lateral ventricular lesion. Pathology confirmed a World Health Organization (WHO) Grade I meningothelial meningioma.

## Discussion

Intraventricular meningiomas were first described by Shaw, et al. in 1854 [2]. Since that time, there have been a number of subsequent series, but due to the rarity of this lesion, reports on large series have remained sparse [1-2]. Surgical routes to the trigone, or atrium, are generally divided into two groups: interhemispheric and transcortical. Transcortical approaches are further divided into transparieto-occipital and transtemporolateral routes. Each approach entails certain risks and benefits, with the optimal approach defined by best access to the long axis of the lesion, early access to tumor blood supply, minimal cortical disruption, the spectrum of the patient’s preoperative neurologic deficit, and the proximity to anatomic structures and pathways [1]. Potential complications include motor weakness, apraxias, aphasias, seizures, visual disturbances, and elements of the Gerstmann syndrome (if approaching on the dominant side), among others [1]. A posterior parietal approach, in theory, can permit good exposure of the tumor with minimal interruption of visual fibers, as anatomical studies have shown that the optic radiation runs inferolaterally to the ventricles and that the ventricular trigone can be reached without interruption of these fibers [1]. However, the significant variability in the course of these fibers necessitates a more individualized approach. In a microsurgical anatomical study of 10 frozen, formalin-fixed human brains, lateral, inferior, and medial approaches were made for optic radiation fiber dissection [3]. The average distance from the tip of the anterior Meyer loop to the calcarine sulcus varied from 95 to 114 mm. The length of the optic radiations varied from 15 to 18 mm. In a similar study, Sincoff, et al. dissected 10 human cadaveric hemispheres to more fully establish the relationship between the optic radiations and the temporal horn [4]. The authors found that traditional lateral approaches to the temporal horn put the visual fibers at risk. Confirming these findings in healthy subjects, Nilsson, et al. reported on several healthy volunteers and two patients with previous temporal lobe resection [5]. DTI was used to visualize the optic radiations and to measure the distances from the edge of Meyer’s loop to certain landmarks in the temporal lobe. The authors found the distance from the most anterior part of Meyer’s loop to the temporal pole to range from 34 to 51 mm and concluded that: 1) Meyer’s loop has a significant variability in its anterior extent, and 2) tractography may be a useful method preoperatively to visualize Meyer’s loop and assess the risk of a visual field defect.

The use of preoperative tractography for surgery via a transcortical route, while implicated in potential improved visual outcomes, has not been widely reported [1]. Furthermore, when reported, sample sizes have been small. In a study of 20 patients undergoing anterior temporal lobe resection, structural MRI scans, DTI, and visual fields were acquired before surgery, and at three to 12 months following surgery [6]. They found that 12 patients (60%) suffered a visual field deficit. Meyer’s loop was predicted from imaging to be 4.4 to 18.7 mm anterior to the resection margin in the patients who were found to have a deficit, but 0 to 17.6 mm behind the resection margin in those without visual field deficit. The variance of the degree of the visual field deficit correlated with the extent of damage to the Meyer’s loop and the authors found good optic radiation accuracy with DTI. They proposed that DTI has the potential to reduce the risk of visual complications. Multiple studies have included tractography as an element in preoperative planning for temporal lobe resection, concluding that DTI is an accurate technique to delineate optic radiation trajectory and will play an increasing role in decreasing postoperative visual performance and disability [6].

In addition to epilepsy surgery, tractography has also been described for safer resections of intracerebral tumors. Multiple studies have investigated the use of white matter tracking during glioma surgery [7-8]. In a series of 37 patients undergoing glioma surgery utilizing preoperative and intraoperative diffusion tensor imaging, Nimsky, et al. found that comparison of preoperative and intraoperative tractography depicted a marked shift of major white matter tracts during glioma removal [7]. The authors noted that the shifting emphasized the need for an intraoperative update of navigation systems during resection of deep-seated tumor portions near eloquent brain areas. While an intricate knowledge of cortical anatomy is essential to safe resection of intracerebral pathology, the added benefit of including advanced methods, such as diffusion tensor imaging to pre- and intraoperative surgical planning, especially in eloquent areas of the brain, is becoming increasingly clear. Periorbital edema is found to obscure standard diffusion tractography, and newer methods have shown greater resolution, especially in the arcuate fasciculus and other tracts in regions of fiber crossing [8]. Yang, et al. reported on using blood oxygen level-dependent functional MRI (fMRI) and diffusion tensor imaging fusion guidance for the resection of small intracerebral lesions in the motor cortex [9]. The authors reported total removal in 12 of 15 cases (80%) and found that this combined fMRI-DTI guidance allowed for a safe, accurate, and effective resection. To our knowledge, there is only one previous paper that has reported on the use of DTI for transcortical resections of intraventricular meningiomas. In 2015, Sun, et al. reported on 60 patients with lateral ventricular meningiomas who underwent diffusion tensor and blood oxygenation level-dependent fMRI for fiber tracking and eloquent cortex localization [10]. The patients were split evenly between study and control groups. The group who underwent fMRI-DTI guidance were found to have a significantly higher rate of visual field preservation than controls (P = 0.01), as well as fewer cases of transient aphasia (P < 0.05).

This study has multiple limitations. As it presents only one example of the use of tractography for the avoidance of optic radiations in the resection of intraventricular meningiomas, no substantial conclusions can be made from this case. Larger controlled studies need to be conducted to reliably present potential changes or additions to surgical practice. There also needs to be a sufficiently powered control group to accurately report improved outcomes.

## Conclusions

Advancements in intra- and preoperative planning will increasingly allow for safer and more effective resections of intracerebral pathology. The addition of enhanced DTI to the neurosurgeon’s armamentarium will only further allow for more complete resections while minimizing complications, such as visual deficit. While a thorough knowledge of cortical anatomy is absolutely essential to safely address these lesions, the significant variability in the fiber bundles, such as the optic radiations, necessitates a more individualized understanding of the patient’s potential risk. While fMRI and stereotactic neuronavigation have become mainstays in surgical resection of brain tumors, enhanced DTI will eventually occupy a similar role. Dependence on methods such as these becomes especially apparent when addressing difficult lesions, such as intraventricular meningiomas.
